# Dual Hydrophilic‐Hydrophobic Core Architecture in Soy Glycinin Amyloid Fibrils Revealed by Cryo‐EM

**DOI:** 10.1002/advs.202509821

**Published:** 2025-08-29

**Authors:** Saiya Li, Shuangjian Li, Yijia Cheng, Yapeng Fang, Qin Cao, Yiping Cao

**Affiliations:** ^1^ Department of Food Science & Engineering School of Agriculture & Biology Shanghai Jiao Tong University Shanghai 200240 China; ^2^ Bio‐X Institutes Key Laboratory for the Genetics of Developmental and Neuropsychiatric Disorders Ministry of Education Shanghai Jiao Tong University Shanghai 200030 China

**Keywords:** cryo‐EM, dual‐core architecture, nanomaterials, soy protein amyloids, structural polymorphism

## Abstract

Plant‐derived amyloid fibrils represent a promising class of sustainable nanomaterials outperforming their native counterparts in functionalities; however, the atomic‐level structural mechanisms behind these enhancements have yet to be elucidated. Using cryo‐EM, near‐atomic resolution structures (3.4 and 3.5 Å) are determined for two distinct fibril polymorphs assembled in vitro from soy glycinin‐A subunit. The dominant Type I fibril exhibits an unprecedented dual‐core architecture, characterized by spatially segregated hydrophilic (Asp172‐Asn178/Asn178’‐Asp172’) and hydrophobic (Val166‐Ile168/Val186’‐Pro184’) domains, which contribute to a unique amyloid fold distinct from many known amyloid structures, including pathological and functional amyloids. In contrast, the minor Type II fibril adopts a conventional extended hydrophobic core with Tyr155‐Tyr158 π‐stacking. These atomic structures establish fundamental structure‐property relationships that will inform the rational design of plant protein‐based nanomaterials.

## Graphical abstract

While high‐resolution structures of animal and microbial amyloids are extensively characterized, the structural principles underlying plant‐derived amyloids remain largely unexplored. The study addresses this fundamental gap in knowledge by determining the first near‐atomic‐resolution cryo‐EM structures of amyloid fibrils derived from soy glycinin, uncovering an unprecedented dual hydrophilic‐hydrophobic core architecture.

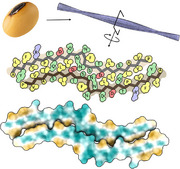

## 1 Introduction

Amyloids, characterized by cross‐β sheet structures, represent a unique class of protein aggregates formed through the ordered self‐assembly of unfolded proteins. While historically associated with neurodegenerative diseases such as Alzheimer's (Aβ) and Parkinson's (α‐synuclein),^[^ ^1^ ^]^ amyloids also serve essential functional roles in biological systems. For example, microbial species employ functional amyloids as structural components in biofilm matrices (e.g., curli fibrils in *Escherichia coli*).^[^ ^2^ ^]^ and as antimicrobial agents (e.g., microcin E492 fibrils in *Klebsiella pneumoniae*).^[^ ^3^ ^]^

Plant‐derived amyloids have recently garnered attention as both functional biological entities^[^ ^4^ ^]^ and sustainable nanomaterials with broad applications.^[^ ^5^ ^]^ These protein assemblies exhibit a remarkable ability to form under diverse conditions, ranging from natural biological contexts to controlled processing environments. Examples include amyloid assemblies with enzymatic regulatory functions in pea storage proteins,^[^ ^4a^ ^]^ as well as the well‐documented amyloidogenic potential of seed storage proteins (e.g., from soybeans,^[^ ^6^ ^]^ peanuts,^[^ ^7^ ^]^ sunflower,^[^ ^7^ ^]^ flaxseeds,^[^ ^8^ ^]^ and almonds.^[^ ^9^ ^]^) under acidic thermal treatment. The resulting fibrils consistently outperform their native precursors in key functional properties, including interfacial stabilization,^[^ ^5a,b^ ^]^ gelation^[^ ^5a^ ^]^ and iron fortification,^[^ ^10^ ^]^ positioning them as promising candidates for future food and biomaterial applications. Despite these advances, the atomic‐level structural basis underlying their enhanced functionality remains elusive. To date, no atomic structures of plant‐derived amyloid fibrils are available in the Protein Data Bank, limiting our ability to rationally engineer these materials for specific applications.

As the most abundant plant‐derived protein, soy protein isolate (SPI) provides an ideal model for structural studies, with glycinin (11S globulin) and β‐conglycinin (7S globulin) as its major components. Cryo‐electron microscopy (cryo‐EM) analysis resolved two distinct fibril polymorphs at 3.4 and 3.5 Å resolution, revealing an unprecedented dual‐core architecture that distinguishes soy protein amyloids from previously characterized pathogenic or functional amyloids.^[^ ^11^ ^]^ These structural insights advance our understanding of soy protein amyloid fibril formation and offer a foundation for their rational design and functional optimization.

## 2 Materials and Methods

### 2.1 Protein Isolation and Fractionation

The isolation and fractionation of protein were adapted from the previous method.^[^ ^6^, ^12^ ^]^ with some modifications. Defatted soybean flour (10% w/v) was suspended in ultrapure water, adjusted to pH 7.5 using 6 m NaOH, and centrifuged (10 000 × g, 20 min). The supernatant was collected and acidified to pH 4.5 for soy protein isolate (SPI) precipitation.

Protein fractionation was performed by sequential precipitation: i) Glycinin fraction was precipitated using NaHSO_3_ treatment (0.98 g L^−1^, pH 6.4, ice bath overnight), and ii) β‐conglycinin fraction was isolated by adjusting supernatant pH to 4.8 and subsequent centrifugation.

**Figure 1:**
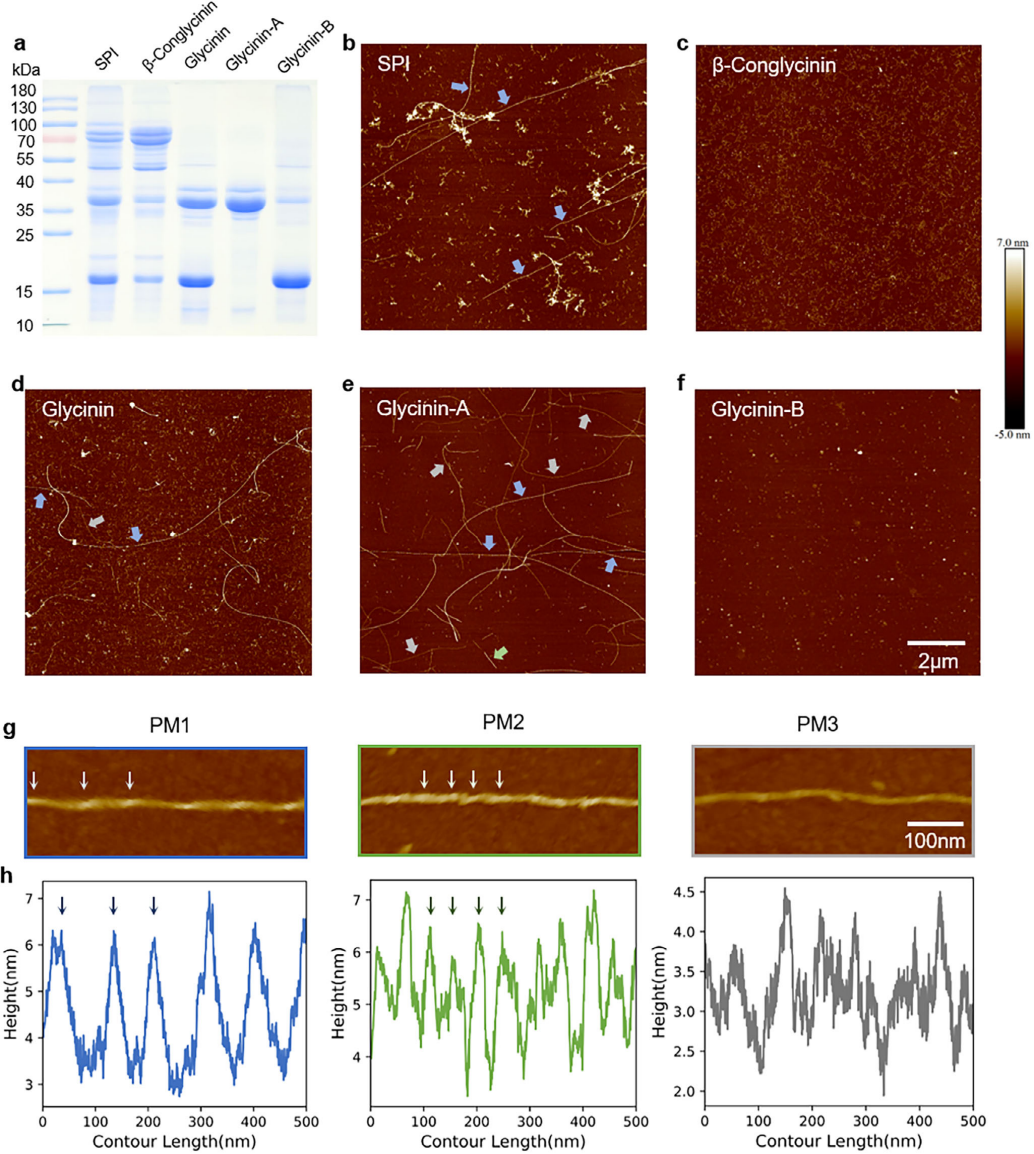
Structural polymorphism of soy protein amyloid fibrils. a) SDS‐PAGE analysis of soybean protein isolate (SPI) and its fractions: β‐conglycinin, glycinin, glycinin‐A, and glycinin‐B. SPI consists of glycinin and β‐conglycinin, while glycinin itself is composed of acidic (glycinin‐A) and basic (glycinin‐B) subunits. (b–f) AFM height images of fibrils formed under denaturing conditions (90 °C, pH 2.0): b) SPI fibrils; c) β‐conglycinin fibrils; d) glycinin fibrils; e) glycinin‐A fibrils; and f) glycinin‐B showing no detectable fibril formation. g) AFM imaging of glycinin‐A fibrils reveals three structural classes: Type I (PM1), Type II (PM2), and Type III (PM3). h) Corresponding height profiles along fibril axes. Arrow color coding: PM1 (blue), PM2 (green), and PM3 (gray).

The glycinin fraction was further dissociated into acidic (glycinin‐A) and basic (glycinin‐B) subunits by heating at 90 °C for 30 min in dissociation buffer (30 mm Tris‐HCl, 15 mm β‐mercaptoethanol, pH 8.0), followed by pH‐dependent precipitation. The resulting protein precipitates were resuspended in ultrapure water, dialyzed (MWCO 3.5 kDa), and lyophilized for further use. A detailed flowchart of the purification procedure is provided in Figure S1 (Supporting Information), and the successful isolation and fractionation of the proteins are demonstrated in **Figure** **1**a. The glycinin‐A used in this study demonstrated >90% purity, as quantified by SDS‐PAGE analysis using ImageJ software.

### 2.2 Sodium Dodecyl Sulphate Polyacrylamide Gel Electrophoresis (SDS‐PAGE)

Protein sample (20 µL, 2 mg mL^−1^) was mixed with 5 µL of protein loading buffer (LT101, Epizyme Biotech). The mixture was vortexed thoroughly, heated at 99 °C in a metal bath for 10 min, and then cooled for subsequent use. SDS‐PAGE gels were prepared using a rapid gel preparation kit (P0012AC, Beyotime Biotechnology), with a 5% stacking gel and a 12% resolving gel concentration. A protein molecular weight marker (10–180 kDa) was used. Samples (10 µL) and marker (3 µL) were loaded onto the gel for electrophoresis (Bio‐Rad, Mini‐PROTEAN Tetra System).

### 2.3 Protein Fibrillization

Protein solutions (20 mg mL^−1^, pH 2.0) were heated at 90 °C for 12 h under constant magnetic stirring (500 rpm) to induce fibril formation. After incubation, samples were immediately cooled on ice and stored at 4 °C for subsequent analysis.

### 2.4 Atomic Force Microscopy (AFM) Imaging and Analysis

The samples were diluted five‐fold with pH 2.0 water, and 20 µL aliquots were deposited onto freshly cleaved mica substrates. Following a 2‐min adsorption, unbound proteins were gently rinsed off using ultrapure water, and the samples were dried under a stream of pure nitrogen gas. AFM imaging was performed using a Bruker Multimode 8 AFM operating in tapping mode with ScanAsyst‐Air probes. FiberApp software.^[^ ^13^ ^]^ was used for quantitative analysis of fibril height profiles and periodicity via autocorrelation functions (ACF) of topographic images.

### 2.5 Negative‐Stain Transmission Electron Microscopy (TEM) Imaging

Fibril samples were prepared for TEM imaging by applying 3.0 µL aliquots of diluted fibril solution (5 mg mL^−1^) onto glow‐discharged 200‐mesh carbon‐coated copper grids (Beijing Zhongjingkeyi, BZ11022a). After 1 min of adsorption, the excess solution was blotted with filter paper. Grids were washed three times with 3 µL ultrapure water and negatively stained with two applications of 3 µL 2% uranyl acetate (10s each), followed by air‐drying for 2 min. The grids were imaged using a Talos L120C G2 TEM (Thermo Fisher Scientific) at 120 kV to confirm fibril morphology and concentration for cryo‐EM data collection.

### 2.6 Cryo‐EM Sample Preparation, Data Collection, and Processing

Cryo‐EM grids were prepared by depositing 3.0 µL of purified fibril solution onto glow‐discharged Quantifoil 1.2/1.3 200‐mesh grids (catalog no. N1‐C14nCu20‐01). Samples were plunge‐frozen in liquid ethane using a Vitrobot Mark IV (Thermo Fisher Scientific). Data were collected on a Titan Krios G3i microscope (Thermo Fisher Scientific) equipped with a Falcon4i Direct Detection Camera (Thermo Fisher Scientific), operated at 300 kV with a 20 eV energy filter. 2274 movies were acquired at a pixel size of 0.932 Å, with a dose rate of ≈1.11 e^−^/Å^2^ per frame across 36 frames, yielding a total dose of ≈40 e^−^/Å^2^. Automated data collection was managed using EPU software.

**Figure 2:**
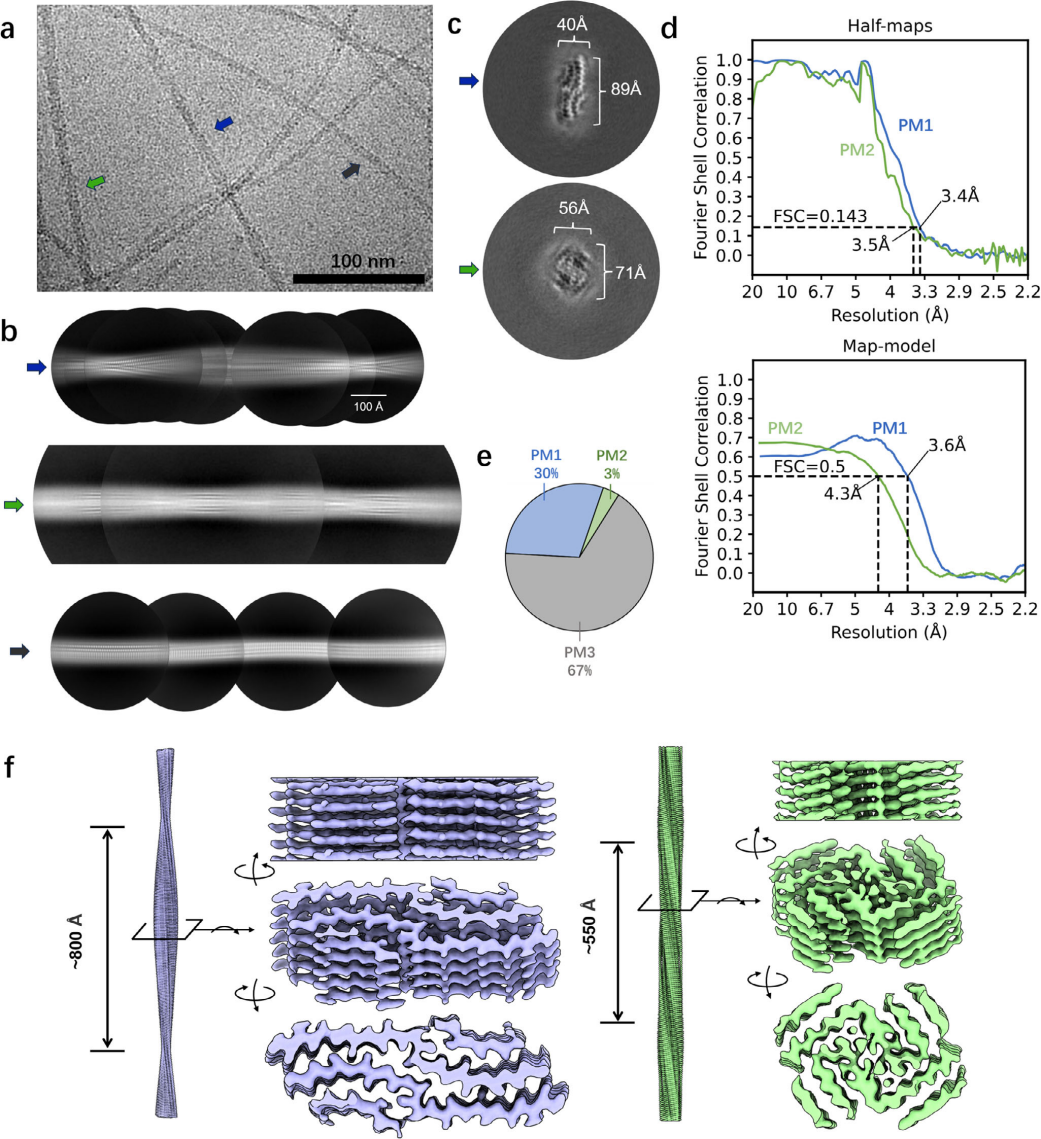
Cryo‐EM structural analysis of glycinin‐A fibrils. a) Representative cryo‐EM micrograph showing the coexistence of three polymorphic populations. b) Representative 2D class averages. c) Cross‐sectional views of the 3D reconstructions for PM1 (89 × 40 Å) and PM2 (71 × 56 Å), with dimensions consistent with AFM measurements. d) Resolution maps for PM1 and PM2 reconstructions. e) Relative abundance of PM1, PM2, PM3. f) Multi‐view representations of PM1 (left panel) and PM2 (right panel).

Data processing (Figure S2, Supporting Information) began with motion correction using MotionCorr2^[^ ^14^ ^]^ and contrast transfer function (CTF) estimation via CTFFIND‐4.1.^[^ ^15^ ^]^ Initial particle picking was manual for 200–300 micrographs, followed by automated picking with Topaz v.0.2.5,^[^ ^16^ ^]^ trained on the manual dataset. Helical reconstruction was performed in RELION 4.0.^[^ ^17^ ^]^ A total of 1228003 particles were extracted using a 360‐pixel box size (10% inter‐box distance), and 364804 particles were extracted with a 1024‐pixel box size (the same inter‐box distance as the 360‐pixel box size). These particles were subjected to 2D classification (tau_fudge = 2), which identified three distinct fibril species (**Figure** **2**b). Helical symmetry was determined through visual inspection of 2D classes (from both 360‐ and 1024‐pixel box sizes) and raw cryo‐EM images. The measurements revealed fibril widths of ≈100 Å with an ≈800 Å pitch for PM1, ≈90 Å with an ≈550 Å pitch for PM2, and ≈65 Å for PM3 (Figure S2, Supporting Information). Initial models were generated through 3D de novo reconstruction. For 3D refinement, particles from selected 3D classes (PM1) or 2D classes (PM2) underwent gold‐standard refinement using the reference model from the 3D de novo reconstruction, achieving resolutions of 3.4 Å (twist 179.466°, rise 2.4 Å) and 3.5 Å (twist −1.6°, rise 4.8 Å), as determined by the 0.143 Fourier Shell Correlation (FSC) cutoff. Data collection and processing statistics are summarized in **Table** **1**.

**Table 1:** Cryo‐EM data collection, refinement, and validation statistics for glycinin‐A PM1 and PM2.

	glycinin‐A PM1 (EMD‐64767, PDB 9V45)	glycinin‐A PM2 (EMD‐64778, PDB 9V4F)
Data collection and processing		
Magnification	×130 000	×130 000
Voltage (kV)	300	300
Electron exposure (e–/Å2)	40	40
Defocus range (µm)	−1.5 – −2.5	−1.5 – −2.5
Pixel size (Å)	0.932	0.932
Symmetry imposed	21	C2
Helical rise (Å)	2.4	4.8
Helical twist (°)	179.466	−1.6
Initial particle images (no.)	1228003	1228003
Final particle images (no.)	71617	14488
Map resolution (Å)	3.4	3.5
FSC threshold	0.143	0.143
Map resolution range (Å)	200‐3.4	200–3.5
Refinement		
Initial model used (PDB code)	De novo	De novo
Model resolution (Å)	3.6	4.3
FSC threshold	0.5	0.5
Model resolution range (Å)	200–3.6	200–4.3
Map sharpening *B* factor (Å2)	129	76
Model composition		
Non‐hydrogen atoms	2140	1990
Protein residues	270	260
Ligands	0	0
*B* factors (Å2)		
Protein	66.52	130.60
Ligand	–	–
R.m.s. deviations		
Bond lengths (Å)	0.004	0.005
Bond angles (°)	1.046	0.973
Validation		
MolProbity score	1.78	2.56
Clashscore	9.62	26.00
Poor rotamers (%)	0	0.43
Ramachandran plot		
Favored (%)	96.00	85.42
Allowed (%)	4.00	14.58
Disallowed (%)	0	0

The third polymorph (PM3), re‐extracted with a 1024‐pixel box, exhibited significant curvature variability, precluding high‐quality reconstruction (Figure S2, Supporting Information).

### 2.7 Atomic Model Building

Two near‐atomic‐resolution cryo‐EM maps (3.4 Å and 3.5 Å for PM1 and PM2, respectively) exhibited clear side‐chain densities after sharpening with phenix.sharpen,^[^ ^18^ ^]^ enabling de novo atomic modeling. Initial models were generated automatically using ModelAngelo 1.0.^[^ ^19^ ^]^ with five candidate sequences of glycinin‐A, achieving robust map fitting for most residues. Models were manually refined in COOT v.0.9.8.1.^[^ ^20^ ^]^ based on the best map‐fitting sequences. A five‐layer model was generated using a Python script and optimized with phenix.real_space_refine (Phenix v. 1.21.1–5286).^[^ ^18^ ^]^ Validation was performed using MolProbity^[^ ^21^ ^]^ (within Phenix), assessing clash scores, Ramachandran outliers, and overall model quality (statistics in Table 1).

## 3 Results and Discussion

### 3.1 Fibrillization Behavior of Soy Proteins

Soy protein isolate (SPI) was extracted from soybean and further fractionated into β‐conglycinin and glycinin, with glycinin subsequently fractionated into glycinin‐A and glycinin‐B (Figure 1a). The molecular weights of these fractions were determined to be ≈70 and 53 kDa for β‐conglycinin, 37 kDa for glycinin‐A, and 18 kDa for glycinin‐B, consistent with a previous report.^[^ ^6^, ^12^ ^]^ SDS‐PAGE analysis confirmed the high purity of the fractionated proteins. Each protein (SPI, β‐conglycinin, glycinin, glycinin‐A, and glycinin‐B) was then subjected to fibrillization under denaturing conditions (90 °C, pH 2.0).

AFM characterization revealed composition‐dependent fibril assembly pathways (Figure 1b–f). SPI formed two morphologically distinct fibril populations: straight fibrils (micrometer length) and curved fibrils (sub‐micrometer length) (Figure 1b). To elucidate the origins of this structural polymorphism, SPI was fractionated into its major components—glycinin (11S globulin) and β‐conglycinin (7S globulin)—and their individual fibrillization behaviors were investigated under identical conditions. β‐Conglycinin formed exclusively curved fibrils (Figure 1c), while glycinin produced both straight and curved fibrils (Figure 1d). Further dissociation of glycinin into its acidic (A) and basic (B) subunits showed that fibrillization was strictly mediated by the acidic subunit (glycinin‐A), which produced well‐defined fibrils, whereas the basic subunit (glycinin‐B) showed no assembly propensity under identical conditions (Figure 1e,f).

High‐resolution AFM imaging of glycinin‐A fibrils revealed three distinct polymorphs (designated PM1, PM2, PM3) (Figure 1g,h) with relative abundances of 27.8%: 1.4%: 70.8%, respectively (Figure S3a,b, Supporting Information). PM1 exhibited classical amyloid characteristics, including left‐handed helicity (90 nm pitch, Figure S3f, Supporting Information) and height variations (5.4 ± 1.5 nm, Figure S3c, Supporting Information). PM2 displayed a different height distribution (6.9 ± 2.0 nm, Figure S3d, Supporting Information), indicative of alternative supramolecular packing. Notably, PM3 fibrils were markedly thinner (3.7 ± 1.5 nm height; Figure S3e, Supporting Information) and did not exhibit clear periodicity in AFM images, potentially representing a distinct polymorph structure compared to PM1 and PM2.

### 3.2 Cryo‐EM Structures of Glycinin‐A Fibrils

The analysis of cryo‐EM micrographs of glycinin‐A amyloid fibrils confirmed the polymorphic diversity initially observed by AFM, revealing three distinct fibril types present in similar proportions (PM1: 30%; PM2: 3%; PM3: 67%) (Figure 2e). Structural heterogeneity hindered the successful reconstruction of PM3 (Figure S2, Supporting Information), while cryo‐EM maps were determined for PM1 (3.4 Å resolution) and PM2 (3.5 Å resolution) fibrils (Figure 2, Table 1).

PM1 exhibited a characteristic twisted architecture with an 800 Å half‐period twist (Figure 2b–f), corresponding to the 90 nm pitch observed by AFM (Figure S3f, Supporting Information). Cross‐sectional analysis revealed dimensions of 89 × 40 Å (Figure 2c), aligning well with AFM height profiles (left panel of Figure 1h). PM2 displayed different cross‐sectional dimensions of 71 × 56 Å (Figure 2c), which remained consistent with the AFM measurements (middle panel of Figure 1h). PM2 fibrils exhibited a shorter half‐period twist of 550 Å (Figure 2b–f). For 3D reconstruction, we applied the 2_1_ symmetry for PM1 and C2 symmetry for PM2.

### 3.3 Atomic Model Building Revealed the Identities of Proteins Constituting the Fibrils

**Figure 3:**
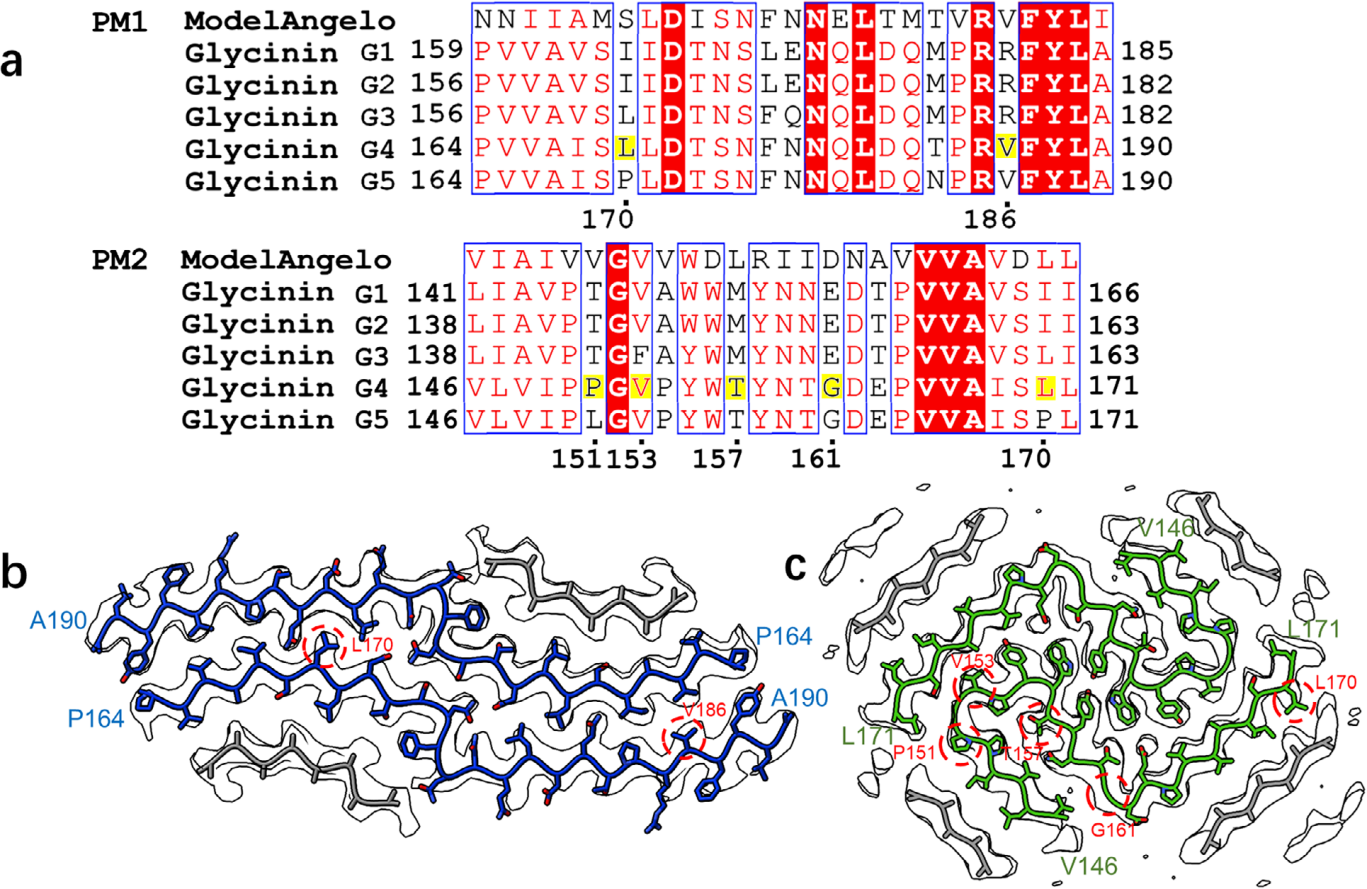
Sequence selection and atomic modeling of glycinin‐A fibril polymorphs. a) Sequence alignment of five candidate glycinin‐A variants (G1–G5) with the ModelAngelo result. Residue numbering follows the glycinin G4 sequence, with key residues for manual inspection indicated. b,c) Cryo‐EM density maps with fitted atomic models for PM1 (b) and PM2 (c). The main chains are highlighted in blue (PM1) and green (PM2), with poly‐alanine fits shown in gray and key residues encircled in red.

To build the atomic model of PM1 and identify the fibril‐constituting protein, we performed de novo model building using ModelAngelo without sequence constraints. The consensus sequence “NNIIAMSLDISNFNNELTMTVRVFYLI” was derived by assigning the highest‐frequency amino acid at each residue position along the parallel β‐sheet layers in the amyloid fibril cryo‐EM map. Sequence alignment of the resulting model against five primary components of glycinin‐A proteins (UniProt IDs: P04776 [G1], P04405 [G2], P11828 [G3], P02858 [G4], P04347 [G5]) revealed optimal matches with two segments: Pro164‐Ala190 of glycinin G4 and G5 (**Figure** **3**a). This observation was corroborated by ModelAngelo's hmm_search algorithm, which independently identified glycinin G4 and G5 as the top‐ranking candidates.

Structural analysis further revealed that Val186 resides within the PM1 dimer interface, where spatial constraints favor small residues (e.g., valine in glycinin G4/G5) but exclude bulkier ones (e.g., arginine in glycinin G1/G2/G3; Figure 3). This strongly supports glycinin G4 and G5 as the fibril‐forming proteins. To discriminate between them, we observed that Leu170 in G4 fits the density map more accurately than Pro170 in G5 (Figure 3b), leading us to select the Pro164‐Ala190 segment of glycinin G4 for final model building.

To build the model for PM2, initial model building was conducted using ModelAngelo, resulting in a model composed of fragmented segments. We manually assembled these fragments based on the main chain trace of the cryo‐EM map to derive the fitted sequence “VIAIVVGVVWDLRIIDNAVVVAVDLL”, and used the sequence of the assembled model for sequence alignment, revealing the highest sequence similarities with Val146‐Leu171 of glycinin G4 and G5, Leu141‐Ile166 of glycinin G1, and Leu138‐Ile163 of glycinin G2 and G3. Among these segments, Val146‐Leu171 of glycinin G4 was chosen as the final segment to build the PM2 model due to the following observations: i) Val153 and Thr157 are embedded in the fibril core without space for large side chains, ruling out glycinin G1, G2, and G3 as candidates due to the presence of large residues at one or both of these positions; ii) Leu170 of glycinin G4 fits the map better than the corresponding Pro170 of glycinin G5; iii) Pro151 and Gly161 are located in kinked regions of the main chain, consistent with their kink‐inducing characteristics.

Comparative modeling further validated our structural findings (Figure S4, Supporting Information). The complete glycinin G4 sequence yielded an excellent map‐model correlation (Figure S4b, Supporting Information). Removing the Pro164–Ala190 segment resulted in markedly poorer fitting (Figure S4c, Supporting Information), underscoring the necessity of this region for structural integrity. Together, these structural and computational analyses consistently identify glycinin G4 as the predominant fibril‐forming protein in both polymorphic forms.

### 3.4 Dual Hydrophilic‐Hydrophobic Core Architecture

**Figure 4:**
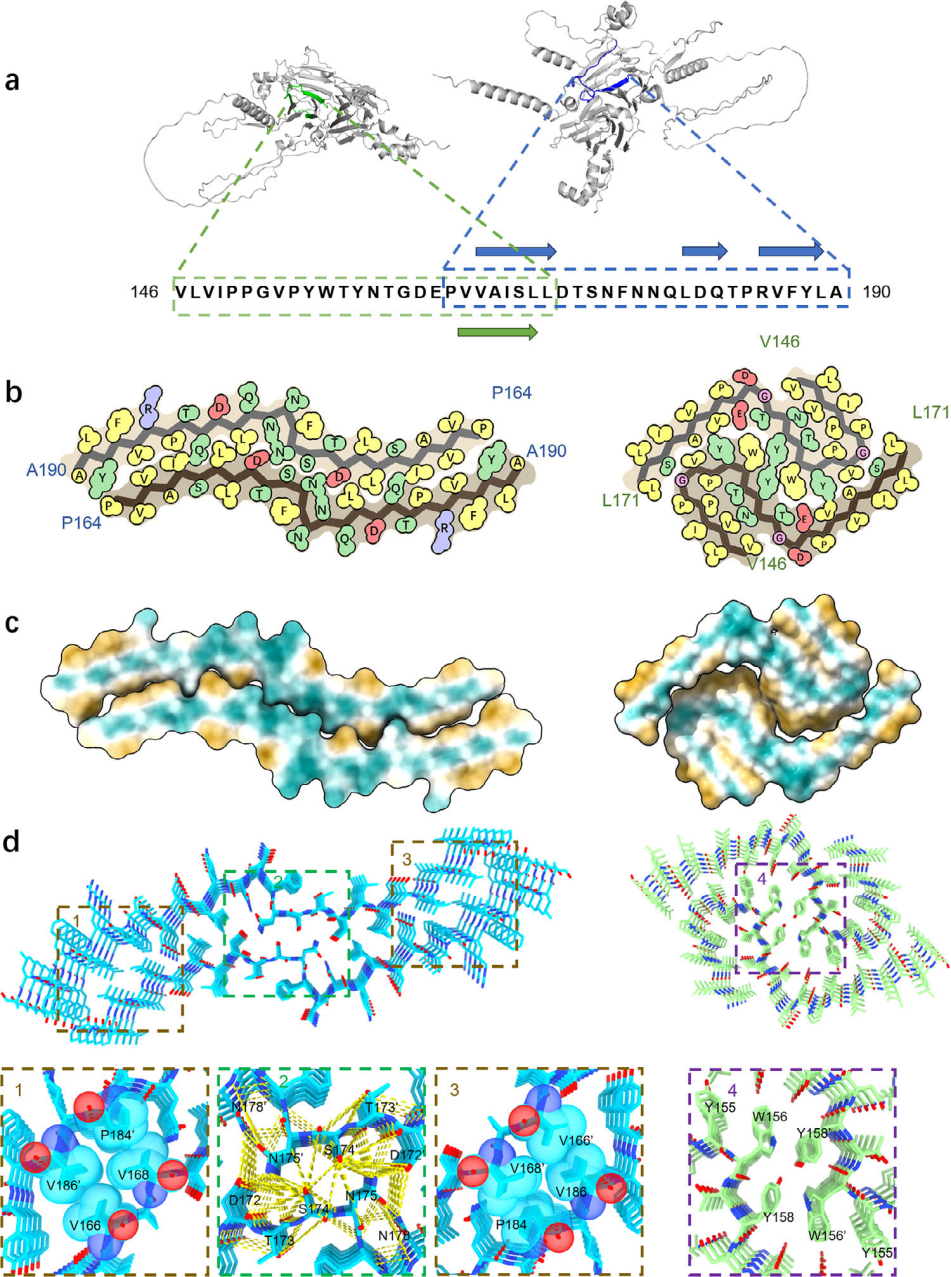
Molecular architectures of PM1 and PM2. a) Primary sequences of PM1 (right) and PM2 (left) are shown in folded glycinin G4 (generated by AlphaFold). b) Cartoon representations of PM1 and PM2 atomic models, color‐coded by residue properties (yellow: hydrophobic; green: polar; red: acidic; blue: basic; pink: glycine). c) Hydrophobicity analysis of PM1 (left) and PM2 (right), with cyan indicating hydrophilic and goldenrod indicating hydrophobic residues. d) Stabilization mechanisms of PM1 and PM2 molecular structures. PM1 exhibits a dual‐core stabilization system comprising: (1–3) two hydrophobic cores and (2) a hydrophilic core with an extensive hydrogen‐bonding network. PM2 stabilization is primarily mediated by (4) a network of π‐stacking interactions.

The two polymorphs exhibit strikingly different structural architectures and stabilization strategies. The PM1 core comprises residues Pro164‐Ala190, with Val166‐Leu171, Leu180‐Gln182, and Arg185‐Leu189 forming β‐strands. Additional densities adjacent to the main chains could not be assigned to specific amino acids because of limited resolution and short chain length. These regions were modeled using poly‐alanine (Figure 3b) for visualization purposes and were not included in the deposited model. An alternative chain conformation of Val165‐Asp172 is shown in Figure S5 (Supporting Information). Importantly, Cryo‐EM analysis of PM1 revealed a structurally unique dual‐core architecture that deviates from conventional amyloid folding patterns (**Figure** **4**). This unique structural motif integrates two stabilization mechanisms: i) a hydrophilic core (Asp172‐Asn178/Asn178’‐Asp172’) forming an extensive hydrogen‐bonded network (≈15 Å diameter), with particularly strong interactions between Asn175, Asn178, and Asp172’ of the opposing chain (Figure 4d), and ii) two hydrophobic core domains (Val166‐Ile168/Val186’‐Pro184’; Val166’‐Ile168’/Val186‐Pro184) featuring steric complementarity with an 8 Å inter‐sheet spacing, preserving β‐sheet registry. This dual‐core architecture represents a significant expansion of known amyloid structural paradigms, demonstrating how plant proteins can evolve alternative stabilization strategies.

In contrast, the PM2 adopts a more conventional amyloid fold stabilized primarily by hydrophobic interactions. The core region (Val146‐Leu171) contains a principal β‐strand (Val165‐Leu170) and characteristic aromatic stacking (Tyr155‐Tyr158) (Figure 4a–d). Due to the limited resolution, additional densities around the main chain could not be assigned to specific peptides, and thus, they were modeled as poly‐alanine strands (Figure 3c) for visualization purposes only and were not included in the deposited model. These unassigned strands in both PM1 and PM2 serve to further stabilize the fibril cores by shielding hydrophobic residues that would otherwise be exposed to the solvent (Figure 3b,c).

### 3.5 Structure‐Function Relationships of Soy Protein Amyloid Fibrils

The atomic structures of glycinin‐A fibril polymorphs elucidate the molecular basis for their functional properties, including iron‐binding capacity,^[^ ^22^ ^]^ and interfacial behavior.^[^ ^23^ ^]^ Although no cysteine residues were identified in the resolved structures, both PM1 and PM2 contain redox‐active aromatic residues (tyrosine in PM1; tyrosine and tryptophan in PM2) likely contributing to the observed iron‐reducing activity. Unresolved fibrils (PM3) may harbor more potent redox sites, such as cysteine.

The unique dual‐core architecture of PM1 fibrils confers exceptional interfacial properties through precisely organized surface domains. Hydrophobic edge residues (Phe187‐Ala190) facilitate adsorption at oil‐water interfaces, while adjacent hydrophilic regions (Asn177‐Arg185) maintain colloidal stability in aqueous environments. This sophisticated amphipathic structure results in a hydrophilic‐lipophilic balance (HLB) superior to native soy protein, explaining the markedly improved emulsification capacity observed in fibrillar systems.^[^ ^23^, ^24^ ^]^

## 4 Discussions

This study determined the near‐atomic resolution cryo‐EM structures of two distinct fibril polymorphs (PM1 and PM2) derived from the glycinin‐A subunit of soy storage protein. Notably, certain peripheral regions in both PM1 and PM2 structures remained unfitted due to limited resolution at the fibril edges. PM3, which constituted the largest proportion, remained unresolved due to significant structural heterogeneity.

PM1 features spatially segregated hydrophilic (Asp172‐Asn178/Asn178’‐Asp172’) and hydrophobic (Val166‐Ile168/Val186’‐Pro184’) domains, a structural motif distinct from previously characterized amyloid folds in both pathological and functional contexts (Figure S6, Supporting Information). This unique provides a molecular basis for the enhanced functional properties of soy protein amyloid fibrils, including their superior interfacial stabilization and iron‐binding capacity. The Asp172 residue, strategically positioned within the hydrophilic core domain, lacks proximal cationic residues. This unusual electrostatic configuration is likely due to its formation under acidic conditions, where the carboxyl group is protonated.

Moreover, despite the remarkable capabilities of advanced artificial intelligence tools like AlphaFold3.^[^ ^25^ ^]^ in predicting protein structures from sequences, their performance in modeling amyloid fibril architectures remains limited. While AlphaFold3 predictions exhibit plausible hydrophilic and hydrophobic patterns, they tend to favor single‐chain spontaneous folding (Figure S7, Supporting Information)—a pattern reminiscent of many disease‐associated amyloid fibrils (Figure S6, Supporting Information). In contrast, our findings demonstrate that PM1 fibrils rely on extensive interchain interactions between their two constituent chains, underscoring the distinctive structural features of this system.

Furthermore, the polymorphism of amyloid fibrils is closely linked to functional heterogeneity—a critical consideration in structural biology. For instance, in vitro‐assembled amyloid beta (Aβ) structures.^[^ ^1b^ ^]^ differ substantially from those isolated from brain tissues,^[^ ^26^ ^]^ a discrepancy that may underlie the clinical failure of therapeutics developed using in vitro Aβ models. In this study, PM1 and PM2 fibrils not only diverge in sequence but also display pronounced structural polymorphism, which likely translates into differences in their functional and material properties. Rather than a limitation, this variability presents an opportunity: the diversity in morphology and composition expands the potential applications of plant‐derived proteins in materials science. The sequence variations enable a broader range of chemical modifications and may confer distinct ligand‐binding capacities, offering a versatile platform for engineering tunable functional biomaterials.

In summary, our findings significantly expand the structural diversity of functional amyloids and establish a framework for the rational engineering of plant protein‐based nanomaterials. The dual‐core architecture of PM1 fibrils, in particular, offers a novel structural paradigm for engineering amphiphilic protein assemblies with tailored applications ranging from interfacial stabilization to antimicrobial surfaces. Looking ahead, future studies should focus on elucidating sequence‐to‐structure relationships across legume storage proteins to predict and control fibril polymorphism and investigating polymorph‐specific biological interactions to unlock the full potential of this sustainable protein nanotechnology platform. By bridging structural insights with functional applications, this work paves the way for the development of next‐generation biomaterials derived from plant‐based amyloid fibrils.

## Conflict Of Interest

The authors declare no conflict of interest.

## Author Contributions

S.L. and Y.C. prepared cryo‐EM grids and collected cryo‐EM data. S.L. and Q.C. processed the cryo‐EM data and built the atomic models. S.L. prepared the protein and fibril samples and contributed to AFM imaging. All authors analyzed the results and participated in writing the manuscript. Y.C., Q.C., and Y.F. supervised the work.

## Supporting information



Supporting Information

Supporting Information

## Data Availability

Cryo‐EM map and atomic model present in this study have been deposited into the Worldwide Protein Data Bank (wwPDB) and the Electron Microscopy Data Bank (EMDB) with accession codes PDB 9V45 and EMD‐64767 for PM1, and PDB 9V4F and EMD‐64778 for PM2. Any other relevant data is available from the corresponding author upon request. The Python script used to generate the five‐layer model is provided in the Supplementary Materials.
