# Supplementary material for: Dual Hydrophilic‐Hydrophobic Core Architecture in Soy Glycinin Amyloid Fibrils Revealed by Cryo‐EM
Source: Adv Sci (Weinh). 2025 Aug 29;12(41):e09821. doi: 10.1002/advs.202509821 (PMC12591115; doi:10.1002/advs.202509821)
Supplement: Supplementary file 1 — Supporting Information [file ADVS-12-e09821-s002.pdf]

Supplementary Materials

Dual Hydrophilic-Hydrophobic Core Architecture in Soy Glycinin Amyloid Fibrils Revealed by Cryo-EM

Saiya Li[1], Shuangjian Li[1], Yijia Cheng[2], Yapeng Fang[1, *], Qin Cao[2, *], Yiping Cao[1, *]

[1] Department of Food Science & Engineering, School of Agriculture & Biology, Shanghai Jiao Tong University, Shanghai 200240, China.

[2] Bio-X Institutes, Key Laboratory for the Genetics of Developmental and Neuropsychiatric Disorders, Ministry of Education, Shanghai Jiao Tong University, Shanghai 200030, China.

* Email: ypfang@sjtu.edu.cn; caoqin@sjtu.edu.cn; caoyiping@sjtu.edu.cn

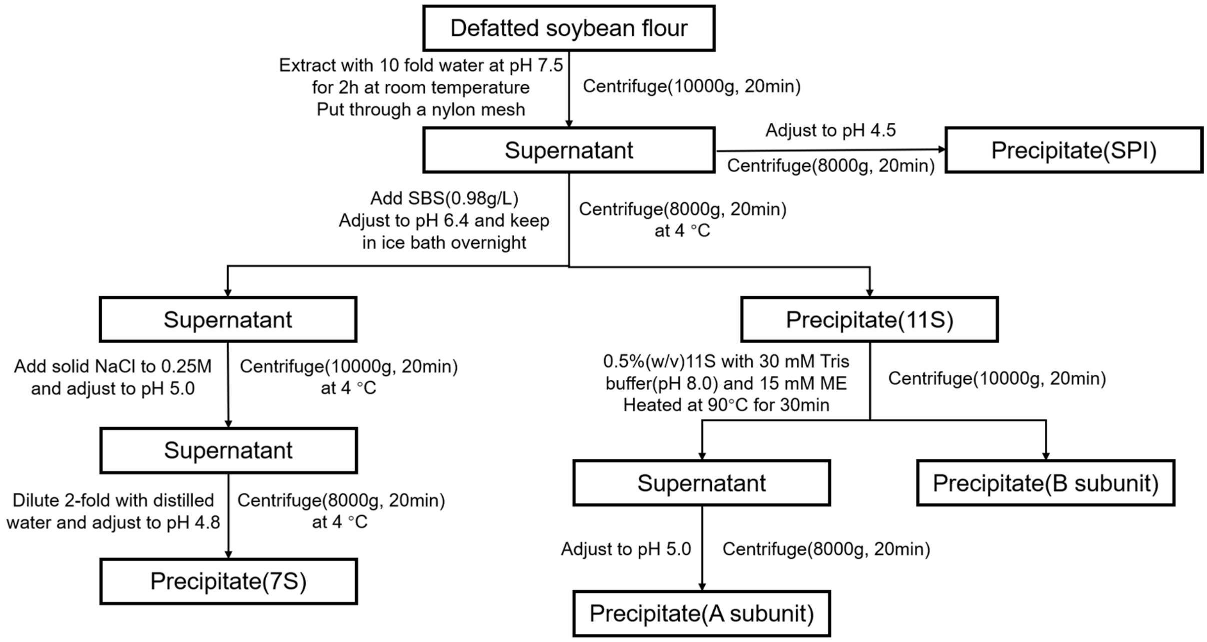

Figure S1. Workflow for the extraction and fractionation of soy protein isolate (SPI) components[1]. The diagram illustrates the sequential isolation of β-conglycinin (7S) and glycinin (11S) fractions, followed by dissociation of glycinin into acidic (A) and basic (B) subunits under reducing conditions.

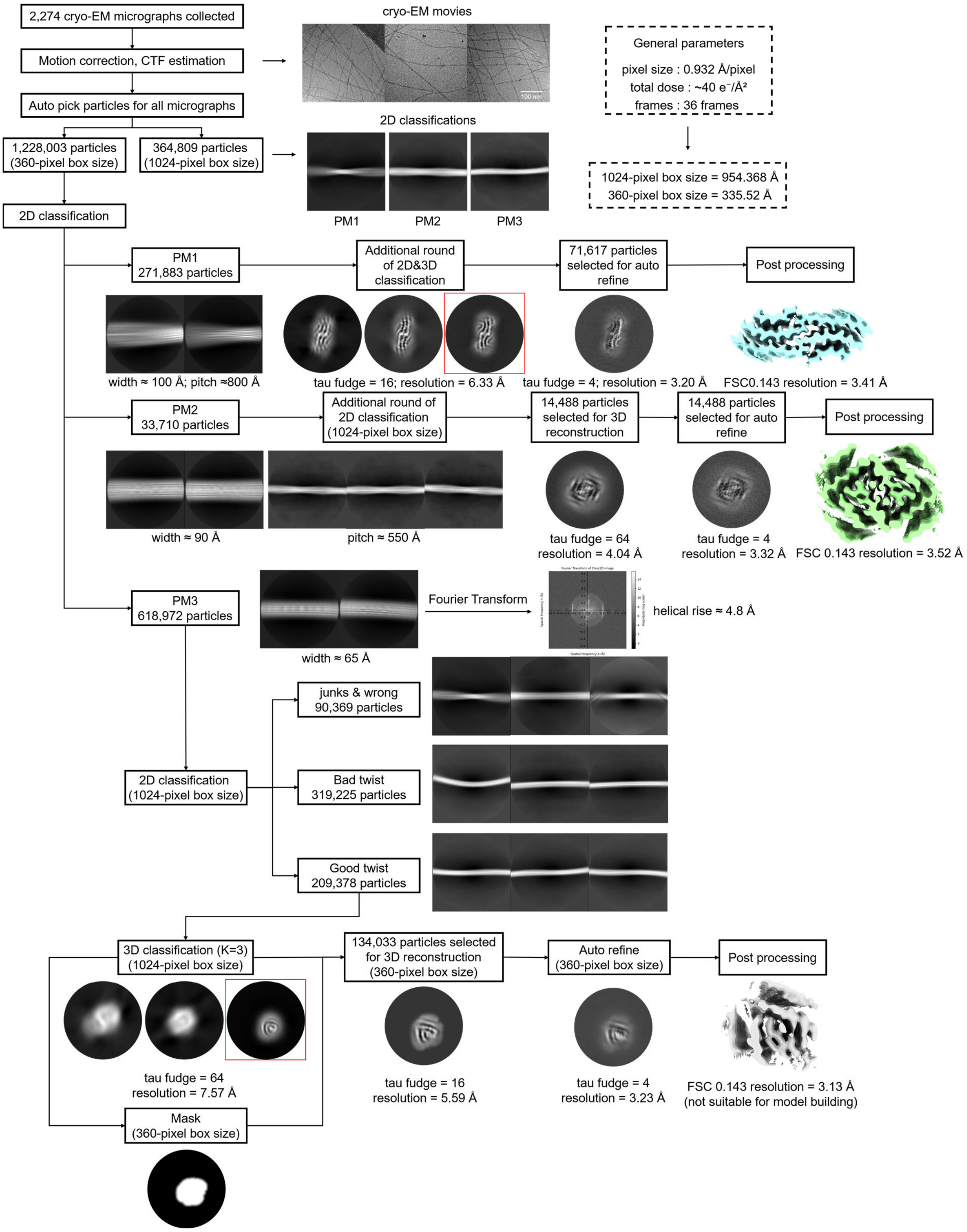

Figure S2. Workflow of cryo-EM data processing.

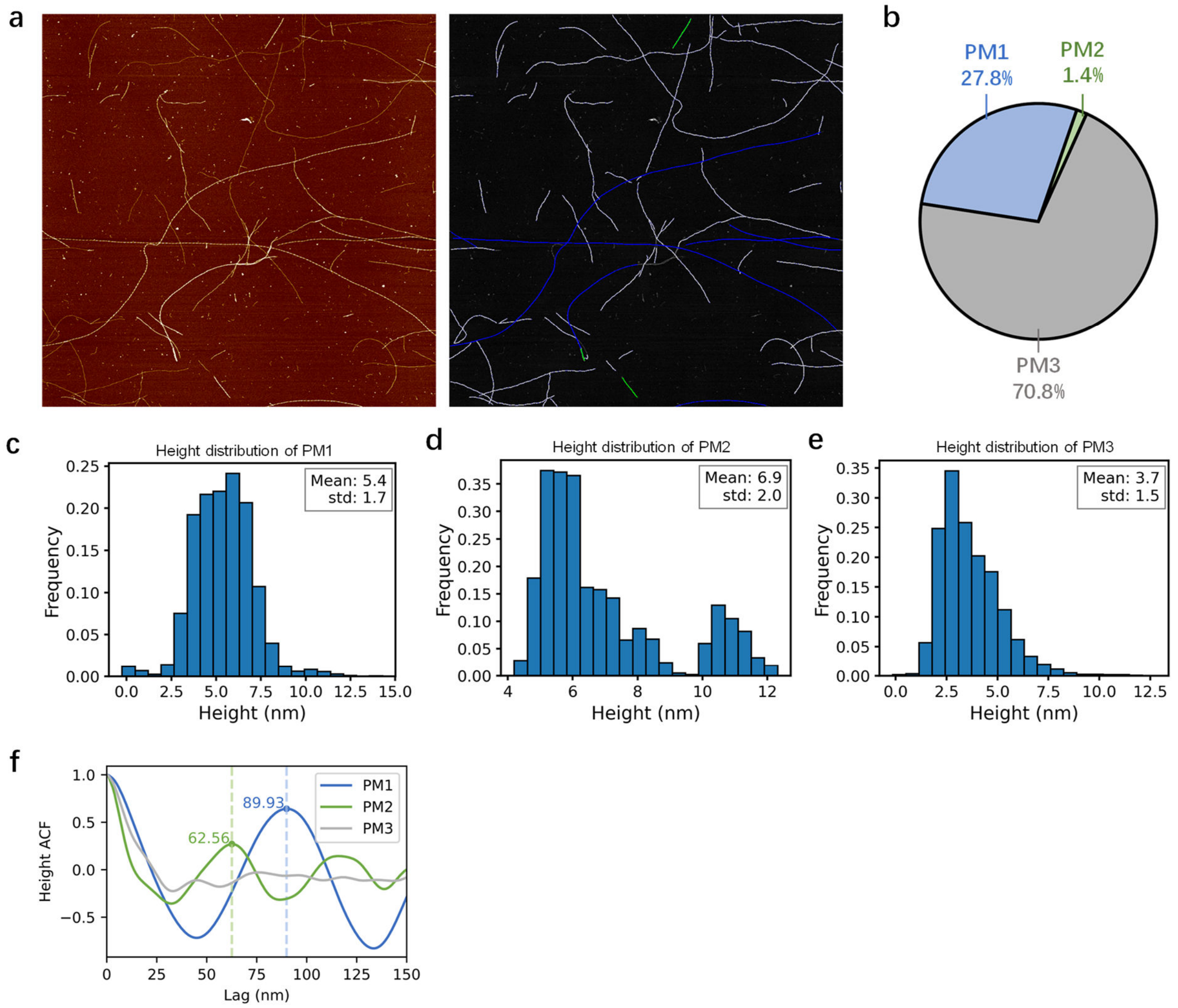

Figure S3. Analysis of glycinin-A fibrils. (a) Identification of PM1, PM2, and PM3 fibrils through manual selection using FiberApp software[2]. (b) Quantitative analysis of polymorph populations based on contour length measurements. (c-e) Height distribution histogram of PM1, PM2 and PM3 fibrils (f) Height autocorrelation function (ACF) analysis revealing periodicities of 89.93 nm (PM1) and 62.56 nm (PM2).

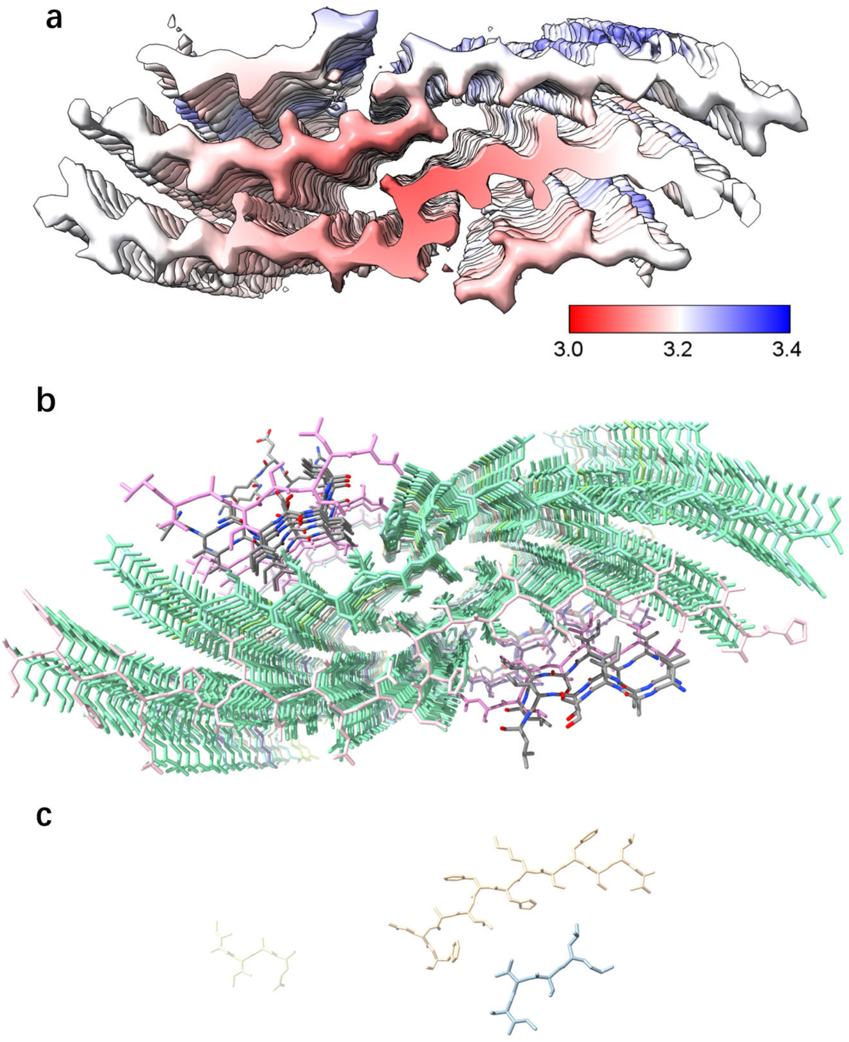

Figure S4. Sequence validation of PM1. (a) Local resolution map of PM1, demonstrating that most regions achieve sub-3.2 Å resolution, enabling reliable atomic modeling. (b) ModelAngelo reconstruction using the complete glycinin G4 sequence, demonstrating excellent density fit for Pro164-Ala190 region. (c) Control reconstruction with the Pro164-Ala190 segment excluded from the input sequence, resulting in significantly poorer map-model fit.

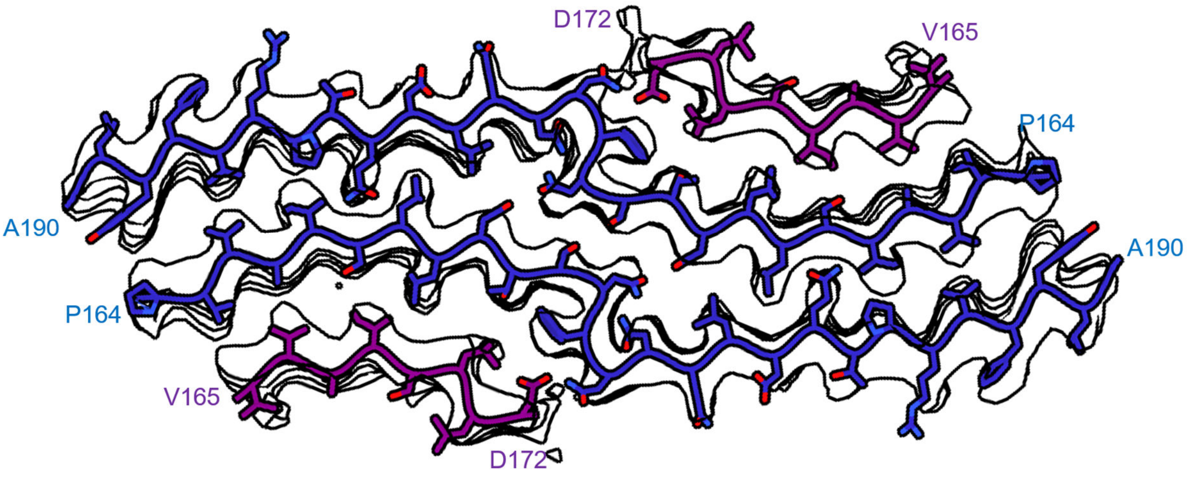

Figure S5. Close-up view of sequence fitting within cryo-EM map. Note that Val165–Asp172 (highlighted in purple) belongs to an adjacent chain, not the Pro164–Ala190 chain.

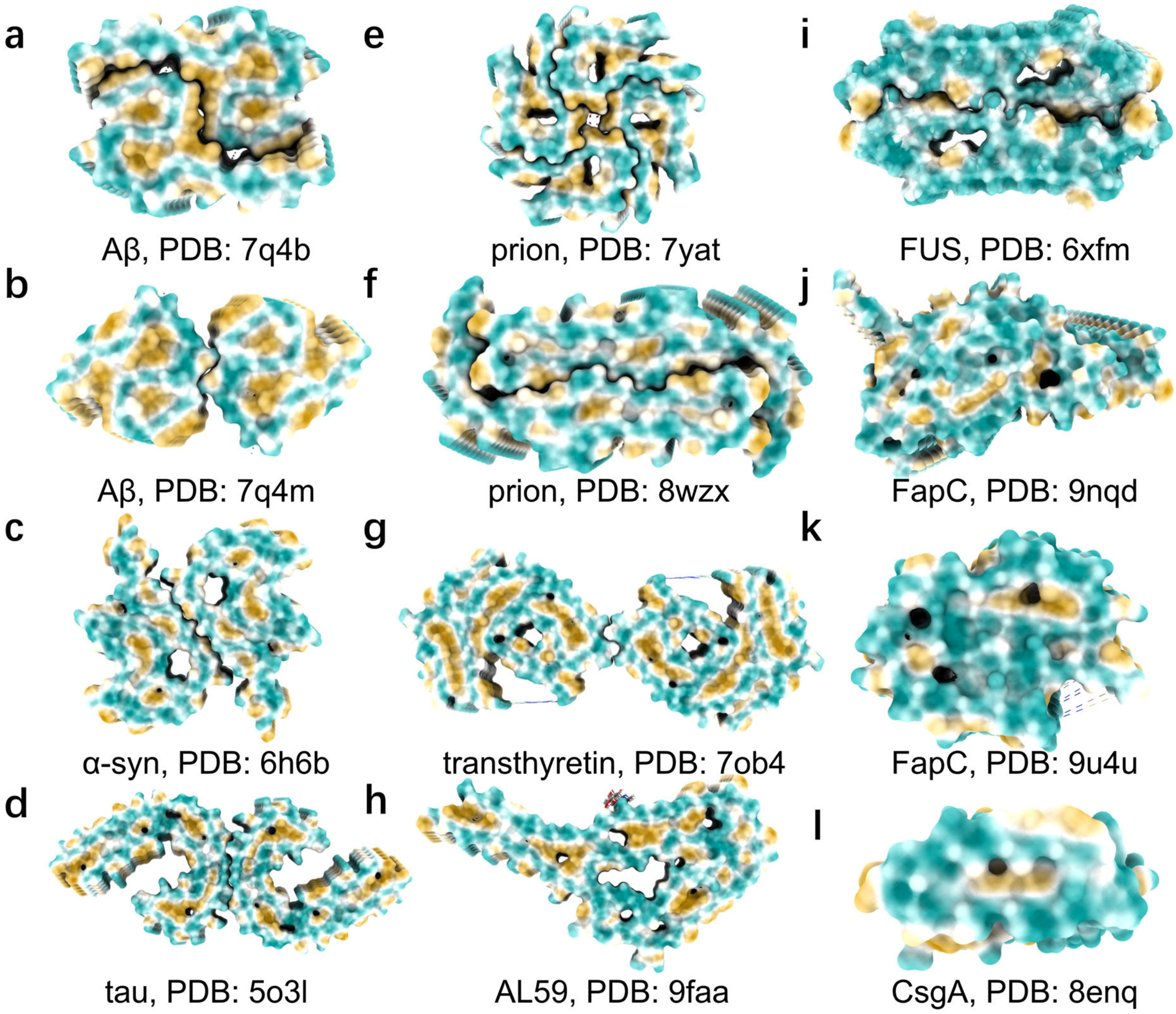

Figure S6. Structural diversity of amyloid fibrils: hydrophobic vs. hydrophilic core architectures. (a–h) Amyloid fibrils stabilized primarily by hydrophobic core interactions. (i–l) Amyloid fibrils featuring predominantly hydrophilic core architectures.

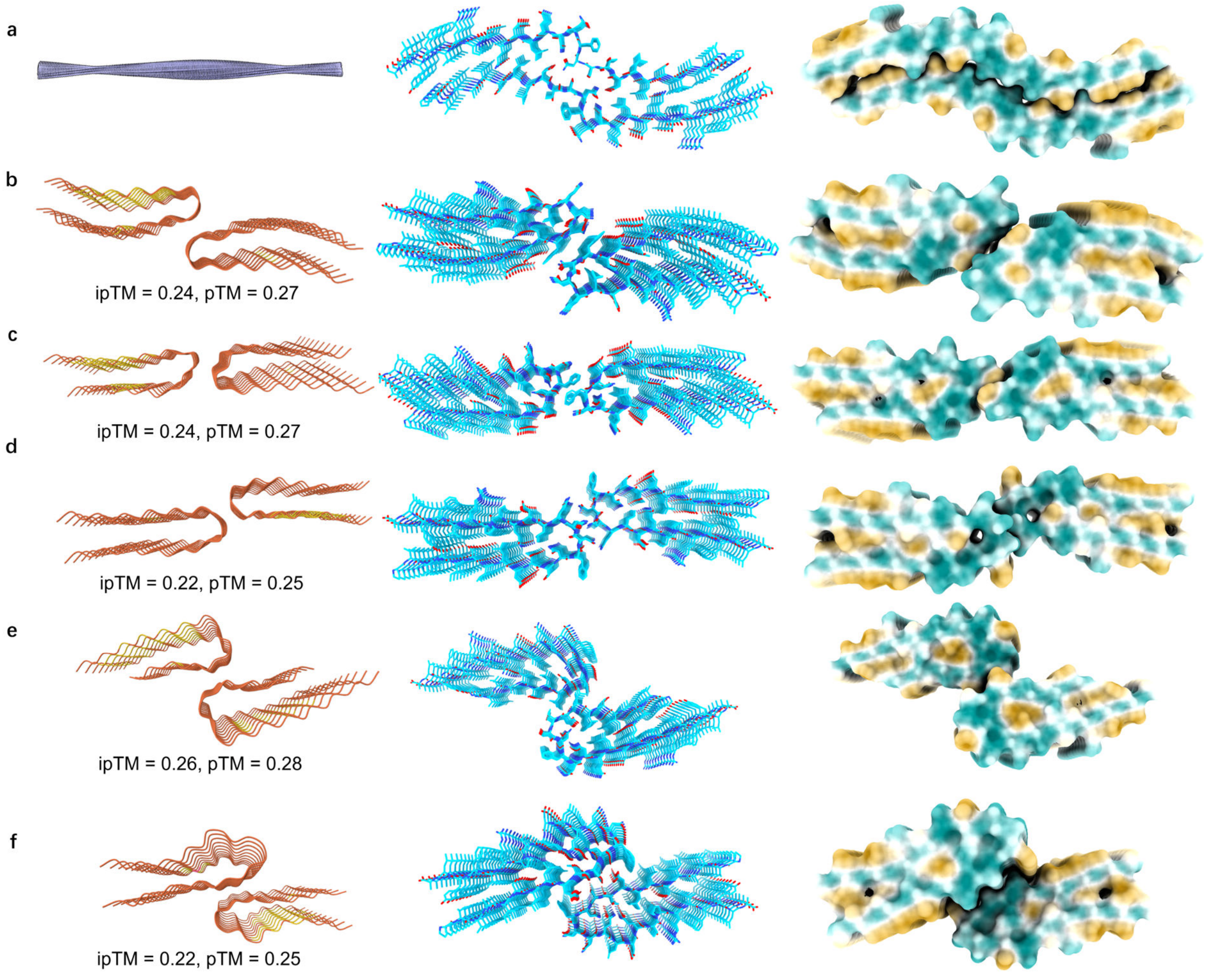

Figure S7. Structure comparison of experiment and alphafold3 prediction [3]. (a) Experimentally determined structure from this study. (b-f) Five independent structural predictions generated by AlphaFold3.

Note: our experimental structure reveals extensive interchain interaction interfaces between the two primary chains, underscoring the current inability of AlphaFold3 to accurately predict the precise packing arrangements in amyloid fibrils. These findings emphasize that, at present, high-resolution structural determination of amyloid fibrils remains reliant on experimental approaches.

[1] a) Y. Zhang, D. R. Dee, *Journal of agricultural and food chemistry* **2023**, *71* (11), 4755; b) T. Nagano, M. Hirotsuka, H. Mori, K. Kohyama, K. Nishinari, *Journal of Agricultural and food chemistry* **1992**, *40* (6), 941.
[2] I. Usov, R. Mezzenga, *Macromolecules* **2015**, *48* (5), 1269, https://doi.org/10.1021/ma502264c.
[3] J. Abramson, J. Adler, J. Dunger, R. Evans, T. Green, A. Pritzel, O. Ronneberger, L. Willmore, A. J. Ballard, J. Bambrick, *Nature* **2024**, *630* (8016), 493.
